# CD73-Dependent Regulation of Interferon AlphaA and Interleukin-10 in the Inflamed Mucosa

**DOI:** 10.1100/tsw.2010.203

**Published:** 2010-11-04

**Authors:** Ilya Sotnikov, Nancy A. Louis

**Affiliations:** Division of Neonatal-Perinatal Medicine, Emory University School of Medicine, Atlanta, Georgia, USA

**Keywords:** CD73, adenosine, nucleotide, interferon alpha, interleukin-10, epithelium, macrophage, dendritic cell, mucosa, intestine, inflammation, HIF1

## Abstract

The ecto-5'-nucleotidase, CD73, catalyzes the rate-limiting step in the phosphohydrolysis of ATP to adenosine, and is a critical regulator of the balance between adenosine and its nucleotide precursors. Each of these classes of mediators signal through their independent receptor families to regulate downstream inflammatory signaling. CD73 activity is primarily regulated at the level of transcription in response to the oxygen-sensing transcription factor HIF1, and its tissue-specific expression correlates negatively with oxygen tension. HIF1-dependent induction of CD73 contributes to the protective effects of hypoxia in the inflamed intestinal mucosa. These beneficial effects of CD73 have largely been attributed to downstream adenosine signaling through its tissue-specific receptors. In addition, adenosine signaling has been directly implicated in the protective effects of hypoxic preconditioning against acute hypoxic or ischemic insults. However, recent work has demonstrated that CD73-/- animals lack the ability to produce interferon (IFN) αA, either at baseline or in response to inflammation. Furthermore, this IFN-αA deficiency is associated with the inability to elaborate interleukin (IL)-10–dependent anti-inflammatory signaling. It remains unclear whether interruption of IFN-αA and IL-10 signaling in the absence of CD73 activity results from a deficiency of its product adenosine or an accumulation of its substrate nucleotides. Current evidence for adenosine- and nucleotide-mediated mechanisms of tissue inflammation is reviewed below (Fig. 1).

## INTESTINAL INFLAMMATION, HYPOXIA, AND PRE-/POSTCONDITIONING

Tissue inflammation is characterized by increased metabolic demands resulting both from up-regulated innate reactive oxygen signaling and from increased oxygen consumption by inflammatory cell infiltrates. At the same time, accompanying edema compromises tissue perfusion, leading to decreased oxygen delivery and tissue acidosis. Ischemia is a cornerstone and a driving force behind intestinal inflammation. Inflammation increases metabolic demands and outstrips the oxygen supply of already poorly perfused intestinal tissue, over-riding its unique adaptations for function under very low oxygen tension.

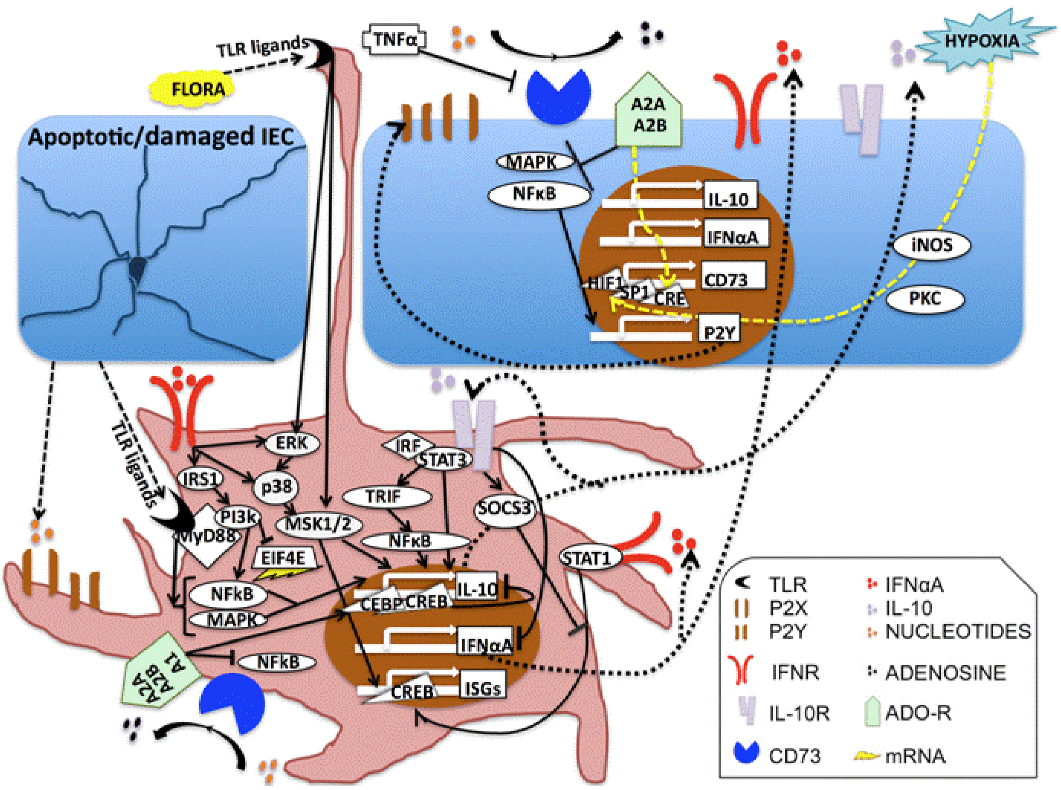

**FIGURE 1.** Overview of hypoxic and inflammatory regulation of CD73-dependent inflammatory signaling in intestinal epithelial cells (IECs) and innate immune cells.

The intestinal epithelium is in fact protected by low oxygen tension through multiple pathways regulated by the key molecular oxygen sensor, HIF1. Specifically, mitochondria serve as major cellular oxygen sensing organelles, generating protective oxygen species necessary for cell survival in hypoxia, driving protective signaling through the stabilization of the transcription factor HIF1[1]. HIF1 is a heterodimeric transcription factor, consisting of constitutively expressed HIF1β and inducible HIF1α protein subunits. In the presence of sufficient tissue oxygen content, the HIF1α subunit is constitutively synthesized, then targeted for degradation by hydroxylation of key proline residues by the prolyl hydroxylase domain enzyme (PHD). In the absence of sufficient molecular oxygen, PHD activity is inhibited, leading to subsequent cytoplasmic accumulation of HIF1α, which then forms a complex with HIF1β and translocates to the nucleus. In the nucleus, the HIF1 complex binds to hypoxia response elements in specific target genes, accountable for cell survival (MDR1), barrier function (MUC3, TFF3), iron metabolism (erythropoietin), and nucleoside metabolism (CD73, A2b) (reviewed in [2]).

Specifically in the intestine, HIF1-mediated transcriptional activation of target genes leads to the elaboration of a series of epithelial responses, which promote baseline barrier function and protect against inflammation-dependent intestinal damage. The loss of epithelial HIF1 expression leads to increased vulnerability to experimental colitis, characterized by increased mortality, weight loss, colonic edema, and increased epithelial permeability. In contrast, overexpression of HIF1 is protective[3].

Tissue responses to inflammation are also critically dependent on hypoxia-dependent regulation of the activity of nuclear factor kappaB (NF-κB). This activation of NF-κB contributes to intestinal protection, playing a key role in cellular survival through the prevention of apoptosis, as well as in the elaboration of inflammatory cytokines[2]. In support of this observation, severe intestinal inflammation develops in mice whose expression of intestinal epithelial IκB kinase-γ has been transgenically knocked out, as well as those deficient in IKKα or IKKβ expression, leading to impaired NF-κB activation[4].

Deficient NF-κB signaling leads to a reduced expression of antibacterial substances, increased epithelial apoptosis, and subsequent increases in microbial translocation through the impaired epithelial barrier. The oxygen dependence of both HIF1 and NF-κB is partially regulated by the same prolyl hydroxylases. This coordinated regulation of NF-κB and HIF1 through their dependence on oxygen tension and proinflammatory mediators define them as pivotal factors in regulating the vulnerability of intestinal epithelia to combined inflammation and ischemia[2].

The critical pathways activated by hypoxia can be exploited in multiple tissues, either to protect or aid in recovery from ischemic insult. Specifically, adaptation to short periods of oxygen deprivation results in tissue protective phenomena, designated either as ischemic preconditioning (IPC) or postconditioning (IPoC), depending on the temporal relationship of the application of the training hypoxic stimulus relative to the acute ischemic episode. Evidence for the protective effects of IPC is found in multiple organs, such as the brain, liver, kidney, and intestine, although much of the work characterizing its underlying mechanisms has been conducted in the myocardium. In fact, IPC was first described in a canine model of acute myocardial infarction in 1986[5]. In these studies, the cardiac muscle was exposed to repeated periods of ischemia and reperfusion, prior to induction of a myocardial infarction. This preconditioning regimen resulted in a significant reduction of infarction size and overall protection to the stressed tissue.

There are two windows of protection that have been described in various species following IPC[6], with early kinase-dependent tissue protection observed during the first 2 h following preconditioning, after which these initial protective effects are no longer evident. Subsequently, a late window of protection begins 24 h after preconditioning and remains in effect for up to 72 h. This later window of protection is dependent on new protein synthesis.

IPC-dependent protective responses involve the activation of the PI3K-Akt axis, production of nitric oxide, reactive oxygen species, signaling by adenosine (Ado), action of mitochondrial ATP-channels, and the formation of mitochondrial permeability transition pores (MPTP). Intriguingly, there is also experimental evidence to support the existence of “remote preconditioning”, in which the introduction of brief episodes of ischemia to limbs results in protective effects on vital organs suffering from acute oxygen deprivation[7].

In contrast, IPoC is induced by the introduction of short intermittent episodes of hypoxia/reoxygenation after an ischemic injury, beginning at the onset of reperfusion[8]. IPoC is crucially dependent on pH in that resultant acidification of the tissue leads to beneficial changes in sodium-hydrogen exchange. In addition, acidification of injured cardiac muscle by perfusion with an acidic buffer alone reproduces the effects of IPoC[9]. The major difference between IPC and IPoC resides in the fact that there is a pronounced inhibition of apoptosis and necrosis in the affected tissues during IPoC[10]. Effects of IPoC are also dependent upon PI3K and ERK1/2 signaling pathways, and culminate with mitochondrial MPTP formation. Ado inoculation at the onset of reperfusion helps to alleviate the damage to the oxygen-starved myocardium by this mechanism, and is associated with decreased inflammatory cell infiltration through a disruption of endothelial interactions with neutrophils[11].

## REGULATION AND ROLES OF CD73

Ado signaling is largely regulated at the extracellular level by the sequential dephosphorylation of ATP, first to AMP, then to Ado, by the respective activities of CD39 and the ecto-5’-nucleotidase, CD73. Importantly, CD73 catalyzes the final and rate-limiting step in this process. CD73 expression is conserved across various species and is well studied in common model systems. While CD73 is expressed on almost every cell type, there is a 50-fold difference between the lowest and highest levels of ecto-5’-nucleotidase activity, with the greatest levels of expression found in the colon, kidney, and brain[12]. CD73 expression is required for the protective actions of Ado in ischemia and inflammation.

While multiple factors regulate CD73 activity, the transcriptional regulation of CD73 by HIF1 appears to dominate and has been extensively characterized (Fig. 2). Microarray RNA expression analysis

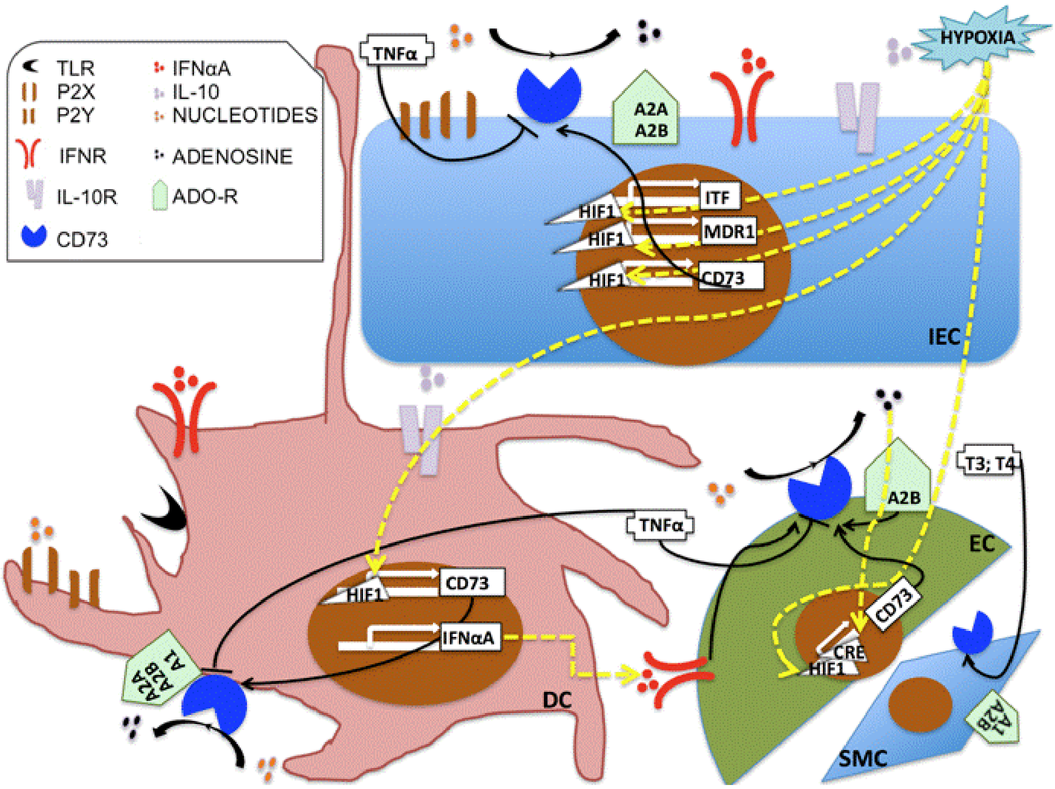

**FIGURE 2.** Regulation of Ado signaling in intestinal mucosa. The cell-specific regulation of CD73 and Ado receptors is depicted in IECs, dendritic cells (DCs), endothelial cells (ECs), and vascular smooth muscle cells (SMCs).

studies demonstrated up-regulation of both CD73 and CD39 mRNA in intestinal epithelial cells subjected to hypoxia. Additionally, a key hypoxia response element was identified within the CD73 promoter[13].

However, DNAse I footprinting analysis of the CD73 promoter also reveals binding sites for CRE (cAMP-response element), Sp1, Sp1/AP-2, LEF-1/TCF, and NF-AT[14]. CRE binding has been implicated in Ado-dependent transcriptional activation of CD73 in microvascular endothelial cells with multiple cAMP agonists transcriptionally activating CD73 expression[15]. However, the significance of these additional transcription factors in the regulation of CD73 transcription remains to be determined.

CD73 expression is also augmented in multiple cell types in a feed-forward fashion through up-regulation by Ado itself, via activation of A2B receptors[15]. This mechanism may contribute to IFNα-dependent CD73 induction and endothelial protection[16].

## Ado SIGNALING

Total Ado levels are governed by the combined effects of extracellular generation of Ado and release from intracellular stores. Ado is released from live cells, as well as cells undergoing apoptosis. Additionally, total Ado levels can be regulated by extracellular Ado inactivation (through conversion to inosine), by Ado deaminase (ADA).

Intact Ado in the interstitial space binds to any of four Ado receptors, A1R, A2AR, A2BR, and A3R (Table 1), which are all expressed on the surface of immune cells. In contrast, epithelial cells express only specific subsets of AdoR. Low-level expression of A1R is demonstrated in the epithelia and subepithelial tissues of the small intestine, while the epithelial cells of the cecum and colon express only the A2BR, and

**TABLE 1**

| Receptor | IEC | Endothelial Cells | Platelets | Neutrophil | Macrophage | pDC | DC |
|---|---|---|---|---|---|---|---|
| CD39 | + | + | + | + | + | +/- | +/- |
| CD73 | + | + | + | - (but + alkaline phosphatase) | +? | +/- | +/- |
| A1 | +low (small intestine) | - | ? | + | - | + | + |
| A2A | - (mouse); + (human) | + | + | + | + | -/low | + |
| A2B | + (cecum, colon) | + | ? | - | + | - | + |
| A3 | + (jejunum, proximal colon) | - | ? | + | + | + | - |
| P2X | + | + | + | + | + | + | + |
| P2Y | + | + | + | + | + | + | + |
| Purine secretion | + | + | + | + | + | ? | ? |
| References | [13,85, 86,87,88, 89,108] | [90,91,92,93] | [85,94,95, 96,97,98] | [90,99,100, 101,102] | [87,90,102,103, 104,105] | [84,85,90, 106,107] | [85,90,106, 107] |

the jejunum and proximal colon are armed with A3R[17]. Depending primarily on the type of G protein coupled to the receptor, different Ado receptors can activate or inhibit downstream cellular signaling (Fig. 3).

It was previously shown that A2A receptors (A2AR) block the production of proinflammatory cytokines and that A2AR agonists attenuate both T-cell–dependent[18] and –independent colitis[19,20]. Additionally, A2AR blockade results in a decrease in the release of the anti-inflammatory cytokine IL-4 from immune cells, and A2AR-deficient mice lack both IL-4 and IL-10[21]. Functions for other Ado receptors have also been implicated in models of inflammatory bowel disease, with overexpression of Ado A2BR reported in experimental colitis[22]. Meanwhile, additional reports of studies in murine models of colitis have indicated a protective role for the Ado A3R[23,24]. Thus, lack of Ado in a setting of intestinal inflammation may be detrimental and, indeed, as it was shown in CD73-/- mice impaired in Ado production, the absence of Ado leads to exacerbation of colitis[25].

During episodes of inflammation, the protective effects of Ado signaling are counterbalanced by the production of proinflammatory cytokines. For example, TNFα, a key cytokine induced in ischemia/reperfusion injury, decreases surface presentation of CD73 and enzymatic activity, which are PLC dependent and MEK/p38 independent[26]. At the same time, TNFα regulates the levels of substrate for the CD39/CD73 axis[27], thus influencing two control points in the protection of endothelial barrier integrity.

In addition to regulation by inflammatory cytokines, CD73 expression and downstream Ado signaling are also driven by critical compensatory responses to tissue ischemia. This regulation is mediated through the hypothalamic-pituitary-thyroid axis, which regulates perfusion of internal organs through changes in blood vessel wall contractility. Thyroid hormone, tri-iodothyronine, T3, which is implicated in vasodilatation, increases CD73 mRNA in vascular muscle cells. At the same time, both, T3 and T4 (thyroxine) treatment increased AMP hydrolysis twofold in the same system[28]. Ado, released as a result

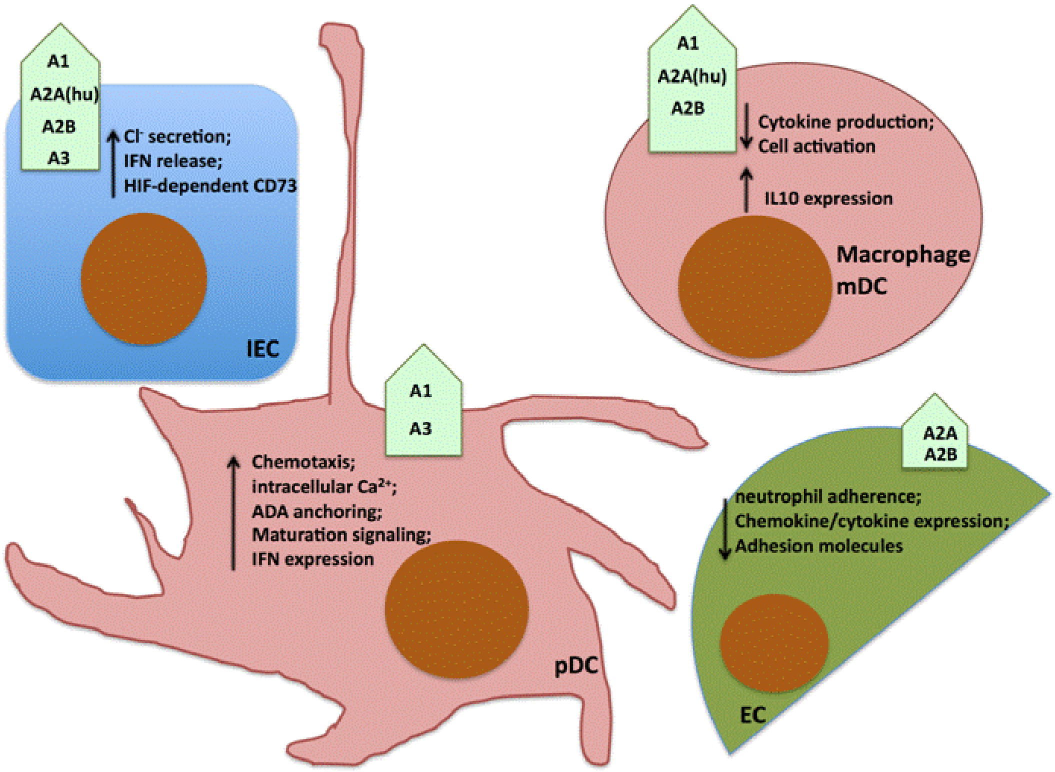

**FIGURE 3.** Cell-specific Ado receptor expression. The cell specific expression of Ado receptors is depicted for IECs, mature DCs (mDCs), plasmacytoid DCs (pDCs), and ECs.

of CD73 activity, acts on A1 and A2B receptors to dilate arteries and increase oxygen supply, thus counteracting the ischemic changes (Fig. 2).

## REGULATION AND ROLES OF TYPE I IFN

Studies in experimental colitis models indicate that CD73 expression is required for the specific expression of IFNαA, a member of the type I IFN family. Type I IFNs include IFNα, IFNβ, IFNω, IFNε, and IFNκ (reviewed in [29]), multifunctional cytokines that play roles in antiviral responses, apoptosis, angiogenesis, and cancer. The majority of nucleated mammalian cells produce specific type I IFNs in response to viruses, bacteria, or Toll-like receptor (TLR) ligands, through activation of IFN response factor (IRF)-3 and IRF-7[30]. These IRFs then activate the type I IFN genes encoded on murine chromosome 4[31] or human chromosome 9[29]. Although many cells are capable of producing type I IFNs, presumably with autocrine or local effects, the predominant tissue source of type I IFNs, *in vivo*, is plasmacytoid DCs (pDCs). This subset of immature dendritic cells (DCs) produces over 1000-fold more cytokine compared to other immune cells (reviewed in [32]).

Upon induction, type I IFNs are released into intracellular spaces where they bind and activate a common receptor consisting of two subunits designated as IFNAR1 and IFNAR2. Chain R1 is constitutively associated with the tyrosine kinase TYK2, while chain R2 has a permanent docking partner, JAK1[33]. These receptor-associated protein kinases then undergo autophosphorylation followed by activation of JAK1and the JAK-STAT pathway[34]. Type I IFNs activate STAT1-6 in a cell-specific manner[35,36,37], leading to the formation of homo- and heteromeric complexes, which translocate to the nucleus to initiate gene transcription by binding to the promoters of IFN-regulated genes (reviewed in [38]).

Regulation of gene transcription and translation by type I IFNs also proceeds in a STAT-independent manner. This involves multiple kinase-dependent pathways, including p38 MAPK-dependent gene activation, through both ISRE and GAS elements in the promoters of IFN-responsive genes[39], as well as via activation of the ERK pathway[40]. Additional signaling mechanisms involve the PI3K pathway through induction of phosphorylation of the docking protein, insulin receptor substrate (IRS) 1, as well as phosphorylation and subsequent activation of p85 subunit of PI3K[41], in a STAT-independent fashion[42]. In a feed-forward manner, PI3K mediates STAT1 activation[43], and enhances NF-κB transcriptional competence[44] and mTOR activity[45]. Through these actions on PI3K and mTOR-dependent phosphorylation, type I IFNs also regulate gene expression through key interactions driving the efficiency of mRNA translation[46].

While it is known that hypoxia induces HIF1-dependent CD73 expression in colonic epithelial cells[13], the role of IFNα in hypoxic tissue is less clear. Under normoxic conditions, IFNα induces HIF1 in a JAK-ISGF3–dependent manner, and this HIF1 up-regulation is linked to IFNα-dependent inhibition of endothelial cell proliferation[47]. It is interesting to note, that IFN-mediated antitumor effects are exerted through induction of microvascular injury and disruption of tumor blood flow, leading to ischemic tumor necrosis[48]. At the same time, hypoxia enhances the antiviral effects of type I IFNs[49]. It seems plausible that regulation of HIF1 is crucial for the functions exerted by type I IFNs, both in normoxia and hypoxia, although a direct mechanistic link between these two mediators has yet to be elucidated.

## CD73, IFNαA, AND COLITIS

However, a seminal paper from our lab provides some evidence linking HIF1-dependent CD73 expression and type I IFN production in inflamed intestinal tissues. Gene array profiling performed on colonic mucosal scrapings of CD73-/- vs. wild-type mice revealed a loss of IFNαA expression in the absence of CD73[25]. It appears that while type I IFNs induce CD73 expression, the activity of CD73, in turn, functions in a feed-forward manner to drive IFNαA expression. Consistent with this finding, inhibition of CD73 hydrolytic activity by α, β-methylene ADP abrogated IFN expression and worsened TNBS colitis in wild-type animals. While treatment with exogenous IFNαA had no effect on disease severity in wild-type mice, it resulted in a partial rescue of TNBS-treated CD73-/- mice, decreasing the severity of disease to a level comparable to that seen in TNBS-treated wild-type controls. One explanation for these findings is that IFNαA treatment functions in the CD73-/- mice to restore a protective mechanism that is already maximally active in wild-type mice.

Another interesting finding in this study was the strict time-dependent up-regulation of IL-10 in wild-type mice with colitis following a transient peak of IFNαA in colonic specimens. This IL-10 response was missing from the CD73-/- mice. The cellular source of IL-10 in this setting remains to be defined. However, the direct involvement of IFNα-related signaling is implied by the finding that the introduction of exogenous IFNαA restored this IL-10 response in CD73-/- mice otherwise lacking detectable IFNαA expression.

Common transcription factor signaling mechanisms may link CD73-dependent effects on IFNαA and IL-10 expression. The transcription factor CEBP, which mediates both type I IFN signaling and IL-10 induction, has also been independently linked to Ado-dependent IL-10 induction in CEBP-deficient macrophages[50]. Additionally, LPS stimulation of macrophages triggered CREB binding to the IL-10 promoter and subsequent IL-10 secretion[51]. Similarly, Ado signaling via A2BR increases IL-10 mRNA stability via 3’-UTR[52]. The roles of similar pathways in IFNα- and Ado-dependent regulation of IL-10 expression may indicate that these pathways cooperate in the induction of IL-10 in colitis. Alternatively, IL-10 induction is of a pivotal importance and, thus, may be regulated by two redundant pathways, one dependent on IFNαA and the other relying on Ado.

Additionally, the regulation of critical IL-10 signaling may occur at the level of RNA stabilization. Indeed, IL-10 mRNA can be stabilized through p38-dependent inhibition of binding of TTP (tristetraprolin, a molecule responsible for rapid degradation of cytokines' mRNA)[53]. Furthermore, a novel method of post-transcriptional regulation of IL-10 expression by microRNA was recently demonstrated. miR-106a, which in turn is regulated by Sp1 and Egr1 transcription factors, inhibits IL-10 mRNA translation[54].

## REGULATION AND ROLES OF IL-10

IL-10 is a ubiquitous cytokine associated with TH2 type responses and, as such, is critical for the successful resolution of intestinal inflammation. IL-10 is produced by numerous cells of the immune system, such as T cells, including TH1, TH2, TH17, and CD8 cells (reviewed in [55]), B cells, NKT cells, eosinophils, neutrophils, mast cells, macrophages, and DCs (reviewed in [56]). Importantly, IL-10 is induced in DCs by ligation of TLR2[57], TLR4 and TLR9[58], as well as in macrophages via TLR3[58]. TLR-independent IL-10 induction also takes place, possibly through DC-SIGN[59] and dectin[60].

TLR-dependent induction of IL-10 occurs through MYD88-dependent MAPKs and NF-κB[61]. It was also demonstrated that LPS-induced IL-10 production in macrophages depends on TRIF (TIR-domain-containing adaptor protein) and may be secondary to type I IFN–dependent regulation of ERK activity[62]. ERK signaling acts as a rate-limiting step for the cell-specific amounts of IL-10 production in subsets of innate immune cells. The highest levels of IL-10 are produced by macrophages (which maximally express ERK), then myeloid DCs (intermediate ERK expression), and negligible amounts of IL-10 are secreted from pDCs (lowest ERK)[63]. MSK1/2-dependent IL-10 production downstream of p38 and ERK was also demonstrated in LPS-stimulated macrophages[51]. Thus, type I IFN may be an ERK-dependent potentiator of IL-10 production in the cells of phagocytic origin.

Interestingly, pDCs as the primary source of IFNs are not potent producers of IL-10. Therefore, during times of peak inflammation, the distribution of tissue-resident innate immune cells shifts towards a predominance of mature macrophages, which elaborate a greater ERK-dependent response to pDC-derived IFNs and secrete IL-10. This shift in innate immune cell populations results in both autocrine and paracrine inhibition of cellular mediators of tissue inflammation. In support of this scenario, TLRs on the surface of cells of the innate immune system induce type I IFNs, which are essential for IL-10 production in an IFNR-, TRIF-, and IRF-dependent fashion[62].

Upon release, IL-10 binds to a heterotetrameric receptor consisting of two subunits unique for IL-10 signaling: IL-10Rα, expressed on cells of hematopoietic origin, and IL-10Rβ, a shared chain utilized by IL-22 and type III IFNs (e.g., IFNλ)[56]. The signaling events induced by IL-10 ligation are based on membrane-proximal activation of Jak1 and Tyk2, and subsequent phosphorylation of STAT3[64]. IL-10–induced STAT3 is linked to the inhibitory functions of IL-10 and in creating the cytokine's autoamplification loop[65]. In regard to STAT signaling, IL-10 was shown to inhibit the STAT1-dependent transcription of IFN-inducible genes in human monocytes[66]. It was also demonstrated that this effect of IL-10 relies on STAT3-independent up-regulation of the suppressor of cytokine signaling (SOCS)-3[67].

This pathway proves to be an important example of a convergence of different cytokine effects utilizing the same signaling molecules. Interestingly, SOCS3 regulates the effects of STAT3 signaling by inhibiting gp130, the receptor of the IL-6 cytokine, where IL-6 exposure in the absence of SOCS3 increases STAT3 and STAT1 signaling with up-regulation of expression of type I IFN–responsive genes[68] and downstream induction of an anti-inflammatory response[69].

## POTENTIAL ROLE FOR NUCLEOTIDE SIGNALING: LESSONS FROM INNATE IMMUNE CELLS

Our discussion thus far has focused on CD73-dependent production of Ado and evidence for its role in the downstream activation of type I IFNs and IL-10. However, in the absence of CD73, nucleotide-mediated signaling may proceed via a different route with nonetheless intriguing outcomes. For instance, intact nucleotides may also signal by binding to specific receptors of metabotropic or ionotropic nature. Murine[70] as well as human DCs[71] express P2 nucleotide-sensing receptors, with human DCs shaping themselves and orienting dendrites towards the source of ATP, suggesting a chemotactic effect of ATP. Moreover, ATP and ATP derivatives, resistant to CD39 hydrolysis, induce endocytosis and up-regulation of CD86, ICAM-1, IL-12 (which were blocked by suramin, a competitive inhibitor of P2 receptors), and MHC Class II expression on peripheral blood–derived human DCs.

Furthermore, P2X receptor activation resulted in synergistic suramin-sensitive effects of ATP on TNFα-dependent up-regulation of the expression of IL-12 and CD83, as well as increased T-cell stimulatory capacity[72]. Murine DCs, on the other hand, are able to respond to UTP in a pertussis-sensitive fashion, suggesting an involvement of P2Y receptor signaling towards inflammatory cytokine production and mobilization of intracellular calcium[73]. The described studies may serve as a plausible explanation for the more pronounced intestinal inflammation, observed in CD73-/- mice, in which signaling via nucleotide receptors is enhanced. However, to complicate matters, another study demonstrated that IL-10 mRNA was up-regulated over 25-fold and IL-10 protein by 12-fold in DCs treated with UTP, relative to unstimulated cells, or cells stimulated with ATP. As mentioned above for ATP, a recent study provides evidence for nucleoside-driven monocyte and macrophage recruitment to the sites of UTP and ATP release from apoptotic cells. This recruitment was dependent on P2Y2 receptor signaling *in vivo*, as demonstrated by RNA interference, specific receptor antagonists, and in P2Y2-null mice[74].

Although some nucleotide receptors display a higher affinity to uridine nucleotides, the effects mediated by P2 receptors may depend on the presence of both ATP and UTP, acting independently from Ado receptors. This evokes cellular responses because hydrolysis of UTP serves to abolish its function[75] and drives the resultant products toward the salvage pathway upon cellular reuptake, as opposed to driving the generation of active species, such as in the case of ATP-AMP axis derivatives. It is possible that while the absence of functional CD39/CD73 in CD73-/- mice attenuates Ado signaling, it simultaneously promotes the P2Y-dependent, UTP-mediated responses. The amount of UTP in cells typically accounts for 10% of the amount of ATP[76], but in the situations of decreased hydrolysis, the levels of extracellular nucleotide may accumulate and actually predominate. The physiological storages of UTP/UDP are present in, and released from, platelets, leukocytes, primary airway epithelial cells, astrocytes, and several cell lines in response to inflammation, tissue damage, or cellular stress[77,78]. UTP is also operative in priming of neutrophils[79], the primary effector cells in intestinal inflammation. Thus nucleotide signaling through purinergic receptors evokes pro- or anti-inflammatory changes in the cells of the immune system, depending on the receptor involved. Studies of ischemia/inflammation delineating the presence of functional nucleotide receptors and their roles in cellular programs are still in their infancy.

## NUCLEOTIDE RECEPTOR SIGNALING IN THE EPITHELIUM

Epithelial cells from colon, lung, and kidney express functional P2Y2, P2Y4, and P2Y6 on the apical membrane[80]. These receptors are implicated in the regulation of cell volume, nonspecific epithelial defense, and ischemic protection (reviewed in [81]). Additionally, P2Y receptor expression is regulated on a transcriptional level by UTP or ATP signaling via a NF-κB response element, concomitantly with PGE2 and Cox-2 secretion in intestinal epithelial cells during inflammation[82]. At the same time, Ado is a negative regulator of NF-κB and MAPK signaling in human intestinal epithelial cells[83]. Thus, in the absence of Ado signaling, which negatively regulates NF-κB, the expression of "anti-inflammatory" P2Y on the surface of epithelial cells may be increased, rendering cells more responsive to UTP.

Interestingly, in human pDCs, UTP (or ATP) binding to P2Y receptors results in a PLCβ/PKC-dependent inhibition of IFNα secretion, brought about by the influenza virus or CpG-A (synthetic TLR9 ligand) exposure[84]. In contrast, TLR7/8-ligand (R-848)–dependent or CPG-B–induced IFNα up-regulation was not affected by nucleotide pretreatment. pDCs do not produce detectable levels of IL-10 in response to external stimuli; thus, they are unlikely to be the source of IFNα-mediated increases of IL-10 in inflammation or hypoxic stress. At the same time, pDCs do respond to IL-10 by decreasing their CpG-triggered production of IL-12, TNF, and IFNα[58]. These findings are of a particular interest in CD73-/- animals where a dramatic decrease in IFNα was observed. Taking into account the complex nature of UTP signaling outcomes in cells of different origin, the prevalent engagement of the P2Y receptor on epithelial cells is hard to predict, although it may explain the observed changes. Studies of the effects of P2Y-dependent signaling IFNαA in the intestinal mucosa may clarify this issue.

## CONCLUDING REMARKS

While it is clear that Ado signaling is critical in tissue responses to hypoxia and plays an important role in mediating the anti-inflammatory effects of the oxygen-sensing transcription factor HIF1, uncertainty remains with respect to the relative contributions of Ado and nucleotide receptor activation in the CD73-dependent regulation of IFNαA and IL-10 expression. Diversity and complexity arise with the tissue- and cell-specific expression of both of these families of receptors. The likely contribution of CD73 to the regulation of mucosal inflammatory responses resides in its role in balancing the levels of Ado relative to total nucleotides present within the mucosa at any given time. Additional studies using whole animal models will be needed to account for the diversity of the cell populations contributing to downstream signaling responses regulated by CD73 in the inflamed mucosa.
